# Inhibitory Potential of Constituents from *Osmanthus fragrans* and Structural Analogues Against Advanced Glycation End Products, α-Amylase, α-Glucosidase, and Oxidative Stress

**DOI:** 10.1038/srep45746

**Published:** 2017-03-31

**Authors:** Ji-Yeon Yang, Jun-Hwan Park, Namhyun Chung, Hoi-Seon Lee

**Affiliations:** 1 Department of Bioenvironmental Chemistry, College of Agricultural and Life Sciences, Chonbuk National University, Jeonju 54896, Korea; 2 Department of Biosystems and Biotechnology, College of Life Sciences and Biotechnology, Korea University, Seoul 02841, Korea

## Abstract

Inhibition of α-amylase and α-glucosidase, advanced glycation end products (AGEs) formation, and oxidative stress by isolated active constituents of *Osmanthus fragrans* flowers (9,12-octadecadienoic acid and 4-(2,6,6-trimethyl-1-cyclohexenyl)-3-buten-2-one) and their structural analogues were evaluated. 9,12-Octadecadienoic acid was 10.02 and 22.21 times more active against α-amylase and α-glucosidase, respectively, than acarbose and ascorbic acid, followed by 9,12,15-octadecatrienoic acid, 9-octadecenoic acid, 4-(2,6,6-trimethyl-1-cyclohexenyl)-3-buten-2-one, 4-(2,6,6-trimethyl-2-cyclohexenyl)-3-buten-2-one, 1-heptadecanecarboxylic acid, and 1-pentadecanecarboxylic acid. Concerning the inhibition of AGEs formation, similar with data for 2,2’-diphenyl-1-picrylhydrazl radical scavenging activities, 9,12-octadecadienoic acid was 3.54 times more active than aminoguanidine, followed by 9,12,15-octadecatrienoic acid, and 9-octadecenoic acid. These results indicate that 4-(2,6,6-trimethyl-1-cyclohexenyl)-3-buten-2-one, 9,12-octadecadienoic acid and their analogues inhibit α-amylase and α-glucosidase, AGEs formation, and oxidative stress have potential value in alleviating diabetic pathological conditions.

Diabetes mellitus (DM) is a metabolic disease that can be accompanied by abnormal plasma blood levels and complications including cardiovascular diseases, neuropathy, and retinopathy^1^. DM affects approximately 2% of the world population^2^. About 90% of those with diabetes have the non-insulin dependent type 2 form (T2 DM)^2^. T2 DM is characterized by relative or complete deficiencies in insulin action and secretion, leading to metabolic disorders and hyperglycemia^2^,^3^. Hyperglycemia can play a leading role as a factor of tissue damage through several mechanisms, including increased flux of glucose and other sugars through the polyol pathway, increased formation of AGEs, increased expression of the AGEs receptor and its activating ligands, activation of protein kinase C isoforms, and over-activity of the hexosamine pathway^4^,^5^,^6^.

Five mechanisms are activated by mitochondrial overproduction of reactive oxygen species^5^. T2 DM associated chronic hyperglycemia can be discerned by examining the postprandial blood glucose level (PBGL)^7^. PBGL has been recently implicated as an important factor in the development and beginning of T2 DM^8^. A sudden rise in PBGLs causing chronic hyperglycemia happens due to the rapid uptake of glucose by intestinal α-glucosidases and hydrolysis of starch by α-amylase. Therapy involves decreasing PBGL by delaying glucose absorption through the inhibition of α-glucosidase and α-amylase, which are carbohydrate hydrolytic enzymes^8^. Inhibition of AGEs formation is another therapeutic option for diabetes that is not dependent on the control of PBGL, and could be useful in the prevention or reduction of diabetic complications. Studies have been performed to develop more effective inhibitors of α-amylase and α-glucosidase, AGEs formation, and oxidative stress from biomaterials to cure diabetes and its complications^4^,^5^,^6^,^8^.

*Osmanthus fragrans* (Oleaceae family) has been domesticated as a local herb in East Asia and is the source of medicinal compounds^9^. *O. fragrans* flowers are also used as additives in foods and beverages^9^, and are considered natural essences and are commonly used in expensive cosmetics and perfumes^9^. *O. fragrans* flowers are used to alleviate pain and coughing, have antioxidant activity, and can provide neuroprotection^10^. Various compounds isolated from *O. fragrans* flowers, including tyrosyl acetate, phillygenin, ligustroside, rutin, and verbascoside findings, indicate that *O. fragrans* flowers may have important pharmacological properties^11^.

Little is known of the potential inhibitory effects of the active constituents isolated from *O. fragrans* flowers on α-amylase and α-glucosidase activities, AGEs formation, and oxidative stress. In this study, the active constituents of *O. fragrans* flowers were identified, and their inhibitory activities were evaluated.

## Results and Discussion

Inhibition of α-amylase and α-glucosidase by the hexane, chloroform, ethyl acetate, butanol, and distilled water fractions partitioned from the methanol extract of *O. fragrans* flowers were evaluated (Table 1). The IC_50_ values for α-amylase and α-glucosidase inhibition were 275.6 and 134.5 μg/mL, respectively. Among the five fractions, the respective IC_50_ value of the chloroform fraction against α-amylase and α-glucosidase was 134.5 and 60.5 μg/mL. The IC_50_ values of the hexane fraction were 250.2 and 120.4 μg/mL, respectively. The inhibitory effect of the chloroform fraction against α-amylase and α-glucosidase was 1.18 and 1.25 times higher than that of the acarbose positive control (IC_50_, 158.4 and 75.5 μg/mL), respectively. A prior study reported strong inhibitory activity (IC_50_ 12.5 μg/mL) of *O. fragrans* extract against α-glucosidase compared with acarbose (IC_50_ 1,081.27 μg/mL)^12^. Treatment with *O. fragrans* extract can decrease PBGL and fasting blood glucose^12^. In the same study, treatment with *O. fragrans* extract (500 mg/kg) significantly decreased the content of serum malondialdehyde and increased the level of superoxide dismutase in diabetic rats, and oral administration of 160 mg/kg of the extract significantly decreased the level of serum triglyceride and serum cholesterol in diabetic rats, and significantly increased liver glycogen content^12^. The present findings bolster the idea that the chloroform fraction derived from *O. fragrans* flowers could efficiently inhibit α-amylase and α-glucosidase, and could possibly play a role in treatment of hypoglycemia through oxidative mechanisms.

**Figure 1:**
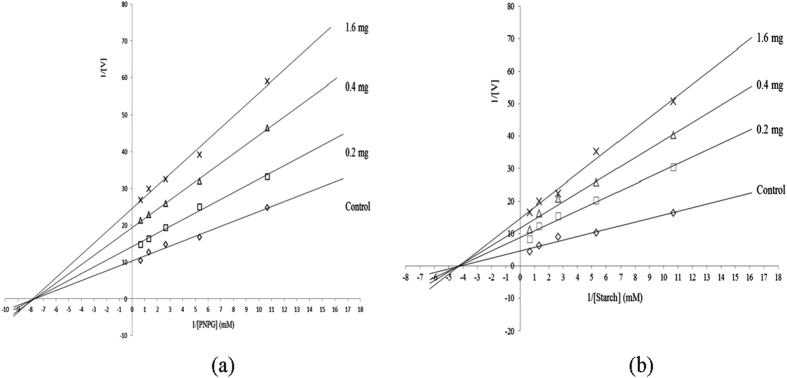
Lineweaver–Burk plot of the inhibition kinetics against α–glucosidase (**a**) and α–amylase (**b**) by the chloroform subfraction subdivided of the methanol extract of *O. fragrans*.

The initial velocity ‘*v*’ of the hydrolysis reactions catalyzed by α-amylase and α-glucosidase was measured using starch or *p*-nitrophenyl-α-D-glucopyranoside (PNPG) as the substrate in the presence and absence of the chloroform fraction (0.2-1.6 mg/mL) are presented in Fig. 1. The regression and extrapolation lines consist of a series of lines crossing on the horizontal and vertical axes. The intercept of the vertical axis (1/Vm) increased as the concentration of the chloroform fraction increased. However, the intercept of the horizontal axis (–1/Km) remained the same. The reaction velocity catalyzed by α-amylase and α-glucosidase slowed and were correlated with an increase in the concentration of the chloroform fraction. The Km values of α-amylase and α-glucosidase were not affected by the concentration of the chloroform fraction, typical of non-competitive inhibition. The results indicate that the chloroform fraction and the substrate did not bind to α-amylase and α-glucosidase at the same site. The data are similar to a prior description of the non-competitive inhibition of α-glucosidase and porcine pancreatic amylase by *Rhus chinensis* extract^13^.

2,2′-Diphenyl-1-picrylhydrazl (DPPH) radical scavenging activities of *O. fragrans* methanol extract and its five fractions were determined (Table 1). The DPPH IC_50_ value of the methanol extract was 69.8 μg/mL. The IC_50_ values of the chloroform, hexane, ethyl acetate, butanol, and distilled water fractions were 60.7, 62.5, 75.2, 76.4, and 129.8 μg/mL, respectively. The inhibitory effects of *O. fragrans* extract and its five fractions against AGEs formation were evaluated by detecting fluorescence; fluorescence intensity of the bovine serum albumin-glucose complex increases with incubation time. The IC_50_ values of *O. fragrans* extract, chloroform, hexane and butanol fractions were 185.8, 110.5, 152.8, and 258.8 μg/mL, respectively. The strength of DPPH radical scavenging and inhibition of AGEs formation were butanol fraction <ethyl acetate fraction <hexane fraction <methanol extract <chloroform fraction. In earlier study, *O. fragrans* extract presented DPPH radical and hydroxyl anion scavenging activity with IC_50_ values of 9.99 and 11.19 μg/mL, respectively^10^. The same study documented the efficiency of *O. fragrans* extract on the ferric reducing/antioxidant power (FRAP) and inhibition of Fe^2+^ chelation with IC_50_ values of 7.74 and 0.23 μg/mL, respectively. The authors described that *O. fragrans* extract reacts with and neutralizes stable free radicals (DPPH and OH radical) independent of any enzymatic activity (FRAP and Fe^2+^ chelating ability). Furthermore, treatment with *O. fragrans* extract can reduce the lipid peroxidation induced by oxidative stress in rat tissues^10^. These prior and the present results indicate that the chloroform subfraction subdivided from the methanol extract of *O. fragrans* flowers may alleviate oxidative stress-related diabetic pathological conditions through the reduction of lipid peroxidation.

The active compounds of the chloroform fraction were isolated using various types of column chromatography and prep HPLC. The chemical structures were identified by EI-MS, ^13^C-NMR, ^1^H-NMR, DEPT-NMR, HMQC and ^1^H-^1^H COSY. EI-MS of OF12 resulted in a signal at *m/z* 280.43, indicating a molecular formula of C_18_H_32_O_2_. The NMR spectra of OF12 are shown in Table 2. In the ^1^H-NMR (600 MHz, CDCl_3_) spectra, 7 distinct peaks were observed. One singlet at δ 9.18 was assigned to the carboxyl (COOH) proton. The signal at δ 2.79–2.83 (t, *J* = 13.6 Hz) was assigned to the methyl (CH_3_) proton. Four signals with two integrated protons (2 H) at δ 3.22-3.28 (m, *J* = 24.4 Hz), δ 3.94-4.00 (q, *J* = 20.8 Hz), δ 4.25-4.29 (t, *J* = 15.2 Hz), and δ 4.68-4.71 (t, *J* = 12.8 Hz) were assigned to the protons attached at alkane (–CH_2_–) and one signal with the integration of one proton (H) at δ 7.28-7.29 (q, *J* = 6.8 Hz) was assigned to the proton attached at alkene (=CH). The ^13^C-NMR (150 MHz, CDCl_3_) spectra showed 18 distinct peaks. A peak at δ 16.39 revealed the methyl functional group (CH_3_). The signal at δ 182.56 was assigned to the acid (–C=O). The signals at δ 24.90, 26.99, 27.97, 29.51, 29.54, 31.36, 31.41, 31.47, 31.68, 31.92, 33.86, and 36.39 were attributed to alkane (–CH_2_–). Other signals at δ 130.24, 130.40, 132.35, and 132.54 were attributed to alkene (C=C). According to the EI-MS and NMR data, this constituent was identified as 9,12-octadecadienoic acid [white liquid, EI-MS, *m/z* 280.43; ^1^H-NMR δ (CDCl_3_) ppm (*J* in Hz): 2.79-2.83 (t, *J* = 13.6 Hz), 3.22-3.28 (m, *J* = 24.4 Hz), 3.94-4.00 (q, *J* = 20.8 Hz), 4.25-4.29 (t, *J* = 15.2 Hz), 4.68-4.71 (t, *J* = 12.8 Hz), 7.28-7.29 (q, *J* = 6.8 Hz), 9.18 (s); ^13^C-NMR δ (CDCl_3_) ppm: 16.39 (C-18), 24.90 (C-17), 26.99 (C-3), 27.97 (C-11), 29.51 (C-14), 29.54 (C-8), 31.36 (C-4), 31.41 (C-5), 31.47 (C-15), 31.68 (C-6), 31.92 (C-7), 33.86 (C-16), 36.39 (C-2), 130.24 (C-10), 130.40 (C-12), 132.35 (C-9), 132.54 (C-13), 182.56 (C-1)]. The NMR data were confirmed from the literature^14^. EI-MS of another fraction designated OF453 resulted in a signal at *m/z* 192.45, indicating a molecular formula of C_13_H_20_O. The NMR spectra of OF453 are shown in Table 3. In the ^1^H-NMR (600 MHz, CDCl_3_) spectra, even distinct peaks were observed. Three singlets at δ 1.26, 1.95, and 2.49 were assigned to the methyl (CH_3_) proton. The signals at δ 2.25–2.28 (t, *J* = 12.4 Hz) and δ 6.29-6.33 (d, *J* = 16.4 Hz) were assigned to the alkene (=CH) proton. The signals with two integrated protons (2H) at δ 1.66-1.68 (t, *J* = 9.2 Hz) and δ 1.79-1.83 (q, *J* = 18.4 Hz) were assigned to the protons attached at alkane (–CH_2_–) and one signal with the integration of one proton (H) at δ 7.45-7.48 (d, *J* = 15.6 Hz) was assigned to the proton attached at the aromatic ring. The ^13^C-NMR (150 MHz, CDCl_3_) spectra showed 12 distinct peaks. The peaks at δ 21.40, 24.25, and 29.68 revealed the methyl functional group (CH_3_). The signal at δ 201.24 was assigned to the ketone (–C=O). The signals at δ 31.32, 36.08, 36.59, and 42.26 were attributed to alkane. Other signals at δ 134.12, 138.46, 138.57, and 145.68 were attributed to alkene (C=C), respectively. According to the EI-MS and NMR data, this compound was identified as 4-(2,6,6-trimethyl-1-cyclohexenyl)-3-buten-2-one [white liquid, EI-MS, *m/z* 192.45; ^1^H-NMR δ (CDCl_3_) ppm (*J* in Hz): 1.26 (s), 1.66-1.68 (t, *J* = 9.2 Hz), 1.79-1.83 (q, *J* = 18.4 Hz), 1.95 (s), 2.25-2.28 (t, *J* = 12.4 Hz), 2.485 (s), 6.29-6.33 (d, *J* = 16.4 Hz), 7.45-7.48 (d, *J* = 15.6 Hz); ^13^C-NMR δ (CDCl_3_) ppm: 21.40 (C-2’), 24.25 (C-6’), 29.68 (C-1), 31.32 (C-4’), 36.08 (C-5’), 36.59 (C-6’), 42.26 (C-1’), 134.12 (C-3’), 138.46 (C-3), 138.57 (C-2’), 145.68 (C-4), 201.24 (C-2)]. The NMR data were confirmed with existing literature^15^.

**Figure 2:**
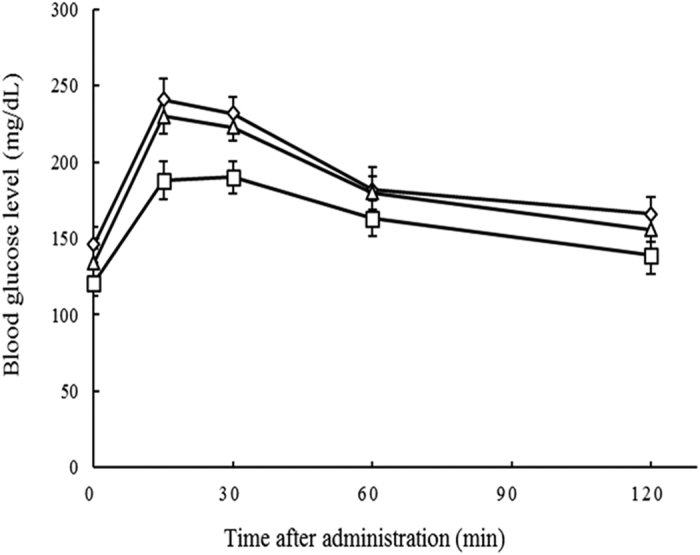
Effects of 4-(2,6,6-trimethyl-1-cyclohexenyl)-3-buten-2-one and 9,12-octadecadienoic acid on blood glucose levels. 2.5 g/kg body weight, with 50 mg/kg with body weight 9,12-octadecadienoic acid (□), 4-(2,6,6-trimethyl-1-cyclohexenyl)-3-buten-2-one (△), and control (◇). Each point represents mean ± SD (*n* = 3).

The inhibitory activities of 4-(2,6,6-trimethyl-1-cyclohexenyl)-3-buten-2-one and 9,12-octadecadienoic acid isolated from *O. fragrans* flowers were measured against α-amylase and α-glucosidase, AGEs formation, and oxidative stress (Table 4). The IC_50_ values of 4-(2,6,6-trimethyl-1-cyclohexenyl)-3-buten-2-one against α-amylase and α-glucosidase were 84.4 and 31.5 μg/mL, respectively. The IC_50_ values of 9,12-octadecadienoic acid against α-amylase and α-glucosidase were 15.8 and 3.4 μg/mL, respectively. Compared with the IC_50_ value of acarbose, which served as a positive control, 4-(2,6,6-trimethyl-1-cyclohexenyl)-3-buten-2-one and 9,12-octadecadienoic acid was 2.40 and 22.21 times more active, respectively, than acarbose (IC_50_ value, 75.5 μg/mL) against α-glucosidase. Against α-amylase, 4-(2,6,6-trimethyl-1-cyclohexenyl)-3-buten-2-one and 9,12-octadecadienoic acid was 1.88 and 10.26 times, respectively, more effective than acarbose (IC_50_ value, 158.4 μg/mL). 4-(2,6,6-Trimethyl-1-cyclohexenyl)-3-buten-2-one and 9,12-octadecadienoic acid significantly inhibit α-glucosidases, which are membrane-bound enzymes secreted in the epithelia of the small intestine and which are crucial for carbohydrate digestion^16^. Inhibition of these enzyme results in a delayed and reduced rise in PBGLs. To confirm that 4-(2,6,6-trimethyl-1-cyclohexenyl)-3-buten-2-one and 9,12-octadecadienoic acid isolated from *O. fragrans* flowers had significant effects on blood glucose levels *in vivo*, a disaccharide loading test was done. Administration of sucrose to fasted mice resulted in a rapid increase in blood glucose concentrations from 146 ± 11.71 to a maximum of 241 ± 13.61 mg/dL after 30 min (Fig. 2). 9,12-Octadecadienoic acid exerted significant suppressive effect on blood glucose levels in mice at 15 and 30 mins compared to sucrose alone. In contrast, 4-(2,6,6-trimethyl-1-cyclohexenyl)-3-buten-2-one showed weak suppressive effect on blood glucose levels at 15 and 30 mins. The results were similar to prior experimental results^17^. These authors assumed that the major components of *Matricaria chamomilla* L., esculetin and quercetin, exerted significant suppressive effect on blood glucose levels in mice. In this regard, our results indicate that 9,12-octadecadienoic acid isolated from *O. fragrans* flowers may alleviate hyperglycemia resulting from high sucrose ingestion by lowering the blood glucose level.

The inhibitory activities of 4-(2,6,6-trimethyl-1-cyclohexenyl)-3-buten-2-one and 9,12-octadecadienoic acid were evaluated by DPPH radical scavenge activity and the inhibition of AGEs formation. These results were compared with those of ascorbic acid and aminoguanidine (Table 4). The DPPH IC_50_ values of 4-(2,6,6-trimethyl-1-cyclohexenyl)-3-buten-2-one and 9,12-octadecadienoic acid were 20.5 and 6.8 μg/mL, respectively. The IC_50_ values of 4-(2,6,6-trimethyl-1-cyclohexenyl)-3-buten-2-one and 9,12-octadecadienoic acid against AGEs formation were 48.4 and 15.4 μg/mL, respectively. Compared with the IC_50_ values of ascorbic acid, which served as a positive control for DPPH radical scavenging, 4-(2,6,6-trimethyl-1-cyclohexenyl)-3-buten-2-one and 9,12-octadecadienoic acid was 1.24 and 3.75 times more active, respectively, than ascorbic acid (IC_50_ value, 25.5 μg/mL). Concerning inhibition of AGEs formation, 4-(2,6,6-trimethyl-1-cyclohexenyl)-3-buten-2-one and 9,12-octadecadienoic acid were 1.13 and 3.54 times more effective, respectively, than aminoguanidine (IC_50_ value, 54.5 μg/mL). 4-(2,6,6-Trimethyl-1-cyclohexenyl)-3-buten-2-one reportedly has anti-proliferative activity and inhibits the cell proliferation of MCF-7 cells by inhibiting Cdk 2 activity^18^. 9,12-Octadecadienoic acid is the precursor contributing to the flavor of mushrooms^19^. A study involving five wild edible mushroom species (*Clitocybe maxima, Catathelasma ventricosum, Stropharia rugoso-annulata, Craterellus cornucopioides*, and *Laccaria amethystea*) comprised of 28.34-74.39% of 9,12-octadecadienoic acid exhibited anti-hyperglycemic and anti-oxidant activities by controlling DPPH radical, α-glucosidase, and hydroxyl free radicals^20^. Presently, 4-(2,6,6-trimethyl-1-cyclohexenyl)-3-buten-2-one and 9,12-octadecadienoic acid isolated from *O. fragrans* flowers significantly inhibited α-amylase and α-glucosidase activities, AGEs formation, and oxidative stress.

**Figure 3:**
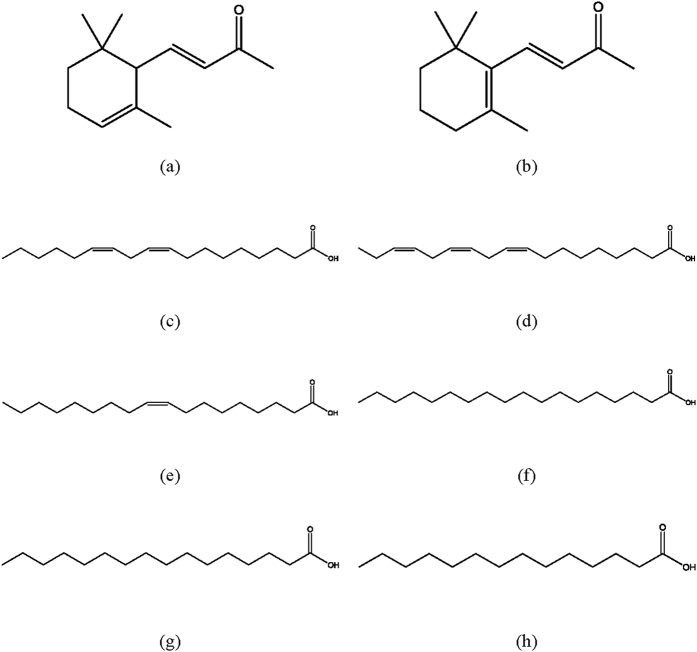
Chemical structures of 4-(2,6,6-trimethyl-1-cyclohexenyl)-3-buten-2-one and 9,12-octadecadienoic acid analogues. (**a**) 4-(2,6,6-trimethyl-2-cyclohexenyl)-3-buten-2-one; (**b**) 4-(2,6,6-trimethyl-1-cyclohexenyl)-3-buten-2-one; (**c**) 9,12-octadecadienoic acid; (**d**) 9,12,15-octadecatrienoic acid; (**e**) 9-octadecenoic acid; (**f**) 1-heptadecanecarboxylic acid; (**g**) 1-pentadecanecarboxylic acid; (**h**) 1-tridecanecarboxylic acid.

To elucidate the structure-activity relationships between the analogues of the isolated constituents and their inhibitory activities, 4-(2,6,6-trimethyl-2-cyclohexenyl)-3-buten-2-one, 9,12,15-octadecatrienoic acid, 9-octadecenoic acid, 1-heptadecanecarboxylic acid, 1-pentadecanecarboxylic acid, and 1-tridecanecarboxylic acid were selected (Fig. 3), and their inhibitory activities were evaluated against α-amylase and α-glucosidase (Table 4). 9,12,15-Octadecatrienoic acid (IC_50_ value, 4.5 μg/mL) was 16.78 times more active than acarbose (75.5 μg/mL) against α-glucosidase, followed by 9-octadecenoic acid (4.8 μg/mL), 4-(2,6,6-trimethyl-2-cyclohexenyl)-3-buten-2-one (33.2 μg/mL), 1-heptadecanecarboxylic acid (46.2 μg/mL), 1-pentadecanecarboxylic acid (55.4 μg/mL), and 1-tridecanecarboxylic acid (60.4 μg/mL). Against α-amylase, 9,12,15-octadecatrienoic acid (21.2 μg/mL) was 7.47 times more active than acarbose (158.4 μg/mL), followed by 9-octadecenoic acid (23.5 μg/mL), 4-(2,6,6-trimethyl-2-cyclohexenyl)-3-buten-2-one (87.2 μg/mL), and 1-heptadecanecarboxylic acid (140.5 μg/mL). 9,12,15-octadecatrienoic acid (IC_50_ value, 7.4 μg/mL) was 3.45 times more active in DPPH radical scavenging than ascorbic acid (25.5 μg/mL), followed by 9-octadecenoic acid (7.6 μg/mL), 1-heptadecanecarboxylic acid (15.6 μg/mL), 1-pentadecanecarboxylic acid (20.8 μg/mL), 4-(2,6,6-trimethyl-2-cyclohexenyl)-3-buten-2-one (23.2 μg/mL), and 1-tridecanecarboxylic acid (24.5 μg/mL). Concerning the inhibition of AGE formation, 9,12,15-octadecatrienoic acid (IC_50_ value, 18.5 μg/mL) was 2.95 times more active than aminoguanidine (54.5 μg/mL), followed by 9-octadecenoic acid (18.8 μg/mL), 1-heptadecanecarboxylic acid (34.7 μg/mL), 1-pentadecanecarboxylic acid (40.6 μg/mL), 4-(2,6,6-trimethyl-2-cyclohexenyl)-3-buten-2-one (50.5 μg/mL), and 1-tridecanecarboxylic acid (51.2 μg/mL). It was previously reported that the inhibitory effects of six pentacyclic triterpenes (corosolic acid, oleanolic acid, maslinic acid, arjunolic acid, asiatic acid and hydroxyursolic acid) against α-glucosidase are affected by the hydroxy group in their carbon 2^21^. In case of flavonoids, the hydroxyl groups at carbons 5 and 7, and the double bond between carbon 2 and carbon 3 have been shown to be important for the strong inhibitory activity on xanthine oxidase^22^. In our study, the inhibitory activities against α-amylase and α-glucosidase activities, AGEs formation, and oxidative stress were affected by the number of carbon chains on the chemical structure, but not by the position and the number of double bonds.

In conclusion, 4-(2,6,6-trimethyl-1-cyclohexenyl)-3-buten-2-one and 9,12-octadecadienoic acid isolated from the methanol extract of *O. fragrans* flowers showed potential inhibitory activities against α-amylase and α-glucosidase activities, AGEs formation, and oxidative stress. These results indicate that 4-(2,6,6-trimethyl-1-cyclohexenyl)-3-buten-2-one and 9,12-octadecadienoic acid exert comprehensive inhibitory effects for preventing related to diabetes and its complications, and have potential in the development of natural preventive or therapeutic agents.

## Materials and Methods

### Chemicals and sample preparation

Acarbose (95%), aminoguanidine hydrochloride (98%), ascorbic acid (98%), bovine serum albumin (98%), DPPH (97%), 1-heptadecanecarboxylic acid (98.5%), PNPG (99%), α-glucosidase (EC 3.2.1.20), 9-octadecenoic acid (99%), 9,12,15-octadecatrienoic acid (99%), porcine pancreatic α-amylase (EC 3.2.1.1, type VI), 1-pentadecanecarboxylic acid (99%), 1-tridecanecarboxylic acid (99%), and 4-(2,6,6-trimethyl-2-cyclohexenyl)-3-buten-2-one (90%) were obtained from Sigma-Aldrich (St. Louis, MO, USA). *O. fragrans* flowers (5 kg) were obtained from a domestic plant store in Jeonju, Korea. A voucher specimen was authenticated by Prof. Kim, Jeongmoon and deposited in Chonbuk National University. *O. fragrans* flowers were extracted with methanol by shaking at 150 rpm at 27 °C for 46 h. Extracts were concentrated with an evaporator at 45 °C^23^. The methanol extract was subdivided based on the polarity of hexane, chloroform, ethyl acetate, butanol, and distilled water. Five fractions were evaporator concentrated, except for the distilled water fraction, which was freeze-dried.

### Isolation and identification of bioactive constituents

To isolate the active constituents from the chloroform subfraction of the methanol extract of *O. fragrans* flowers, column chromatography (4 cm i.d.×60 cm L.) was conducted with silica gel. Ethyl acetate and methanol were the elution solvents. The flow rate was 4.9 ml/min. The chromatographic analysis yielded five fractions (OF1-OF5) were obtained. OF1 and OF4 were subjected to chromatography using a Sephadex LH-20 column (GE Healthcare, Schenectady, NY, USA). From OF1, five fractions (OF11-OF15) were obtained. OF12 was isolated by prep HPLC as a single peak. From OF4, six fractions (OF41-OF46) were obtained. OF45 was isolated using a Jaigel-W253 column (2 cm i.d.×50 cm L., JAI Co., Tokyo, Japan) with 100% methanol at a flow rate of 2.9 mL/min, resulting in the five fractions (OF451-OF455). OF453 was examined further.

Mass spectra (*m/z*) were obtained by mass spectrometry using a QP-2010 quadrupole device (Shimadzu Co., Kyoto, Japan) equipped with an electrospray source, operating in electron ionization (EI) mode at 70 Ev^16^. Mass spectrometry (MS) parameters were as follows: negative ionization mode, capillary, 2.89 kV; column temperature, 51 °C and gradually increased to 209 °C; source temperature, 250 °C; and mass range, 10-300 eV. The chemical structures of the isolated active compounds were analyzed in CDCl_3_ on a JNM EX-600 spectrometer (JEOL Co., Tokyo, Japan). ^1^H (600 MHz), ^13^C (150 MHz), and DEPT (100 MHz) NMR were measured. 2D NMR (^1^H–^1^H COSY and HMQC) was performed to study the relationships between protons and carbons. Tetramethylsilane was used as a standard. The chemical shift was described as δ (ppm).

### α-Glucosidase inhibition

The inhibitory activity of each sample against α-glucosidase was measured as previously described^24^. Sample (25 μL) and phosphate buffer (25 μL; PB, 100 mM and pH 6.8) containing α-glucosidase (0.2 U/mL) was preincubated in a 96 well plate at 37 °C for 10 min. After 10 min, 5 mM PNPG (50 μL) in 100 mM PB was added and incubated at 37 °C for 15 min. The reaction was stopped by adding 150 μL of 200 mM NaCO_3_. Absorbance at 405 nm was recorded with a SpectraMax^®^ microplate reader (Molecular Devices, Sunnyvale, CA, USA) and compared with the control contained 25 μL of 100 mM PB in place of the sample.

### α-Amylase inhibition

The inhibitory activity of each sample against α-amylase was measured as described previously^8^. Sample (40 μL) dissolved in sodium phosphate buffer (SPB, 20 mM and pH 6.9) with 6 mM NaCl was added to 1.0 U/ml α-amylase (200 μL) in SPB and incubated at 30 °C for 10 min. After 10 min, 400 μL of 0.3% starch solution in SPB was added to each tube. This reaction was carried out at 37 °C for 10 min and stopped with the treatment of reagent (100 μL) consisting of 1% 3,5-dinitrosalicylic acid, 12% sodium potassium tartrate, and 400 mM NaOH. Each tube was incubated in boiling water for 20 min and cooled to 27 °C. The reaction was diluted by adding 10 ml distilled water and absorbance at 540 nm was measured using a UV-Vis spectrophotometer. The control contained 20 mM SPB (pH 6.9, 200 μL) instead of α-amylase.

### Inhibitory kinetics against α-glucosidase and α-amylase

The inhibition mode of the chloroform subfraction from the methanol extract of *O. fragrans* flowers against α-glucosidase and α-amylase was evaluated with increasing concentrations of PNPG or starch as the substrate in the absence or presence of the chloroform fraction at a concentration of 0.2, 0.4, or 1.6 mg. The inhibition type was determined by Lineweaver-Burk plot analysis of the data, calculated from the results using Michaelis-Menten kinetics.

### DPPH radical scavenging activity

DPPH radical scavenging activity of each sample for the inhibition of diabetic complications was evaluated as described previously^25^,^26^. Methanol solution (100 μL) containing some concentrations of the sample or ascorbic acid (0.2 mg/mL) as a positive control was mixed with 0.2 mM DPPH (100 μL) in wells of a 96-well microplate. Each reaction volume was mixed and allowed to stand in the dark at 28 °C for 30 min. After the reaction time, the absorbance change of the resulting solution was measured at 517 nm with the aforementioned SpectraMax^®^ microplate reader. The DPPH solution was freshly prepared daily, and was covered and kept in the dark at 4 °C until the measurements were made. Measurements were performed at least in triplicate.

### Inhibition of AGEs formation

The inhibitory activity against AGEs formation was measured as described previously^27^. To prepare the AGEs reaction solution, bovine serum albumin (10 mg/mL) dissolved in 50 mM SPB (pH 7.4) was added to 200 mM fructose, 200 mM glucose, and 0.02% NaN_3_ to prevent the growth of bacteria. The reaction solution (950 μL) was mixed with various concentrations of the samples (50 μL) in 10% (CH_3_)_2_SO. After incubation at 37 °C for 7 days, the fluorescence intensity of the reaction solution was analyzed using a spectrofluorometric detector (Bio-Tek Inc., Winooski, VT, USA) with an excitation wavelength of 350 nm and an emission wavelength of 450 nm. Aminoguanidine hydrochloride served as a positive control.

### Disaccharide loading test

After an overnight fast, male mice (31-35 g) were used for acute disaccharide loading tests (Fig. 2). Disaccharide (sucrose, 2.5 g/kg body weight) as well as the test samples (50 mg/kg of body weight for 4-(2,6,6-trimethyl-1-cyclohexenyl)-3-buten-2-one and 9,12-octadecadienoic acid) were dissolved in 0.9% NaCl solution and administered to the mice via a stomach tube. A control group was loaded with sucrose only. The blood glucose levels were measured using an Accu-Check Performa portable kit, (Roche Diagnostics Korea Co., Ltd., Seoul, Korea).

### Statistical analysis

The IC_50_ value of each sample is expressed as mean ± standard error (SE). Variance analysis for each sample was performed using one-way ANOVA. The significance of differences was obtained at a level of p < 0.05 using SAS 9.2 version software (SAS Institute Inc., Cary, NC, USA).

## Additional Information

**How to cite this article:** Yang, J.-Y. *et al*. Inhibitory Potential of Constituents from *Osmanthus fragrans* and Structural Analogues Against Advanced Glycation End Products, α-Amylase, α-Glucosidase, and Oxidative Stress. *Sci. Rep.* **7**, 45746; doi: 10.1038/srep45746 (2017).

**Publisher's note:** Springer Nature remains neutral with regard to jurisdictional claims in published maps and institutional affiliations.

## Figures and Tables

**Table 1:** IC_50_ values of five fractions partitioned from the methanol extract of *O. fragrans*.

Materials	IC_50_ values (μg/ml, means ± SE)			
	AGE	α–Amylase	DPPH	α–Glucosidase
Methanol extract	185.8 ± 1.6	275.6 ± 2.1	69.8 ± 2.0	134.5 ± 1.9
Hexane fraction	152.8 ± 1.8	250.2 ± 2.1	62.5 ± 1.4	120.4 ± 2.3
Chloroform fraction	110.5 ± 2.5	134.5 ± 1.7	60.7 ± 2.1	60.5 ± 1.6
Ethyl acetate fraction	258.8 ± 1.7	290.4 ± 1.9	75.2 ± 0.5	143.5 ± 2.1
Butanol fraction	>500	325.5 ± 1.2	76.4 ± 0.6	184.3 ± 2.3
Distilled water fraction	>500	0	129.8 ± 1.2	0
Acarbose		158.4 ± 1.4		75.5 ± 1.8
Ascorbic acid			25.5 ± 0.4	
Aminoguanidine	54.5 ± 0.7			

Acarbose was used as the positive control for α–glucosidase and α–amylase. Ascorbic acid was the positive control for DPPH. Aminoguanidine was the positive control for the inhibition of AGEs formation.

**Table 2:** ^1^H- and ^13^C-NMR spectra of OF12.

Carbon	Partial structure	δ_C_ (ppm)	δ_H_ (ppm)
1	COOH	182.56	9.18 (s)
2	C-2H	36.39	4.25-4.29 (t, *J* = 15.2 Hz)
3	C-2H	26.99	3.22-3.28 (m, *J* = 24.4 Hz)
4	C-2H	31.36	3.22-3.28 (m, *J* = 24.4 Hz)
5	C-2H	31.41	3.22-3.28 (m, *J* = 24.4 Hz)
6	C-2H	31.68	3.22-3.28 (m, *J* = 24.4 Hz)
7	C-2H	31.92	3.22-3.28 (m, *J* = 24.4 Hz)
8	C-2H	29.54	3.94-4.00 (q, *J* = 20.8 Hz)
9	C-H	132.35	7.28-7.29 (q, *J* = 6.8 Hz)
10	C-H	130.24	7.28-7.29 (q, *J* = 6.8 Hz)
11	C-2H	27.97	4.68-4.71 (t, *J* = 12.8 Hz)
12	C-H	130.40	7.28-7.29 (q, *J* = 6.8 Hz)
13	C-H	132.54	7.28-7.29 (q, *J* = 6.8 Hz)
14	C-2H	29.51	3.94-4.00 (q, *J* = 20.8 Hz)
15	C-2H	31.47	3.22-3.28 (m, *J* = 24.4 Hz)
16	C-2H	33.86	3.22-3.28 (m, *J* = 24.4 Hz)
17	C-2H	24.90	3.22-3.28 (m, *J* = 24.4 Hz)
18	C-3H	16.39	2.79-2.83 (t, *J* = 13.6 Hz)

s, singlet; t, triplet; m, multilet; q, quartlet.

**Table 3:** ^1^H- and ^13^C-NMR spectra of OF453.

Carbon	Partial structure	δ_C_ (ppm)	δ_H_ (ppm)
1	C-3H	29.68	2.485 (s)
2	C	201.24	
3	C-H	138.46	6.29-6.33 (d, *J* = 16.4 Hz)
4	C-H	145.68	2.25-2.28 (t, *J* = 12.4 Hz)
1’	C-H	42.26	7.45-7.48 (d, *J* = 15.6 Hz)
2’	C	138.57	
	C-3H	21.40	1.95 (s)
3’	C-H	134.12	2.25-2.28 (t, *J* = 12.4 Hz)
4’	C-2H	31.32	1.79-1.83 (q, *J* = 18.4 Hz)
5’	C-2H	36.08	1.66-1.68 (t, *J* = 9.2 Hz)
6’	C	36.59	
	C-3H	24.25	1.26 (s)

s, singlet; d, doublet; t, triplet; q, quartlet.

**Table 4:** IC_50_ values of isolated active constituents and their structural analogues.

Chemicals	IC_50_ values (μg/ml, means ± SE)			
	AGE	α–Amylase	DPPH	α–Glucosidase
4-(2,6,6-Trimethyl-2-cyclohexenyl)-3-buten-2-one	50.5 ± 1.8	87.2 ± 1.3	23.2 ± 2.5	33.2 ± 1.8
4-(2,6,6-Trimethyl-1-cyclohexenyl)-3-buten-2-one	48.4 ± 2.3	84.4 ± 0.8	20.5 ± 1.5	31.5 ± 2.2
9,12-Octadecadienoic acid	15.4 ± 1.8	15.8 ± 2.3	6.8 ± 1.2	3.4 ± 1.5
9,12,15-Octadecatrienoic acid	18.2 ± 2.3	21.2 ± 3.6	7.4 ± 0.9	4.5 ± 2.4
9-Octadecenoic acid	18.8 ± 1.1	23.5 ± 1.8	7.6 ± 1.5	4.8 ± 0.9
1-Heptadecanecarboxylic acid	34.7 ± 2.6	140.5 ± 2.3	15.6 ± 2.2	46.2 ± 2.5
1-Pentadecanecarboxylic acid	40.6 ± 0.7	168.2 ± 1.4	20.8 ± 1.3	55.4 ± 3.2
1-Tridecanecarboxylic acid	51.2 ± 1.8	225.2 ± 2.3	24.5 ± 1.8	60.4 ± 1.2
Acarbose		158.4 ± 1.4		75.5 ± 1.8
Ascorbic acid			25.5 ± 0.4	
Aminoguanidine	54.5 ± 0.7			

Acarbose served as the positive control for α–glucosidase and α–amylase. Ascorbic acid was the positive control for DPPH. Aminoguanidine was the positive control for the inhibition of AGEs formation.
